# The Behavior-Driven Mechanism of Consumer Participation in “Carbon Neutrality”: Based on the Promotion of Replacing Coal with Biomass Briquette Fuel

**DOI:** 10.3390/ijerph192215133

**Published:** 2022-11-16

**Authors:** Qiang Wang, Wenhao Song, Xi Peng

**Affiliations:** 1 School of Business, Shandong Management University, Jinan 250357, China; 2 Jinan Ailing Information Technology Co., Ltd., Jinan 250101, China; 3 School of Humanities, Shandong Management University, Jinan 250357, China; 4 School of Finance, Chongqing Technology and Business University, Chongqing 400067, China

**Keywords:** biomass briquette fuel (BBF), willingness to consume (WTC), willingness to spend (WTS)

## Abstract

“replacing coal with biomass briquette fuel” can effectively reduce carbon emissions. This study takes this as an example to discuss consumers’ “willingness to consume (WTC)”, “willingness to spend (WTS)” and related influencing factors to find the behavior-driven mechanism of consumer participation in “carbon neutrality”. Through the survey and analysis, the results show that 81.64% of the respondents support to consume Biomass Briquette Fuel (BBF) to replace coal. The annual WTS is 157.78 CNY per capita. The factors, such as the education, the relevant government policy support cognition, the level of cognition of health concepts, ecological environmental protection and resource regeneration, have a significant positive impact on the promotion in rural areas. Finally, we put forward corresponding policy recommendations. It provides a reference for motivating consumers to participate in “carbon neutrality” and promoting rural energy transformation to achieve the goal of “carbon neutrality”.

## 1. Introduction

Under China’s “Carbon Peak, Carbon Neutrality” strategy (referred to as the “Double Carbon” strategy), the development of renewable energy is one of the important solutions. As a renewable energy with stability and chemical energy storage properties, biomass energy is one of the high-quality options in renewable energy [1]. Promoting the replacement of fossil energy by biomass energy is a high-quality method to realize the low-carbon transformation of energy. It plays an important role in the “Double Carbon” strategy. The most economical biomass fuel is Biomass Briquette Fuel (BBF) [2]. BBF is a directly combustible clean fuel processed from agricultural and forestry wastes as raw materials. BBF can effectively reduce NOx and SOx emissions in the process of utilization, and has good social benefits and emission reduction potential [3].

The utilization of BBF not only has a huge potential for emission reduction and carbon sequestration, but is also an inevitable requirement for improving the rural living environment. Rural biomass waste represented by straw has the characteristics of wide distribution and stable supply in the vast rural areas [4]. It is a huge potential for rural carbon reduction and carbon sequestration. However, it also faces the problem of environmental pollution due to improper disposal [5]. In order to meet the people’s growing demand for a better life, the green transformation and development of energy in rural areas has become a major part of building a rural energy system [6]. The annual output of crop straw in China is as high as 900 million tons, of which about 200 million tons is unused [7]. The improper treatment and inefficient use of these biomass resources have caused serious impacts on urban and rural ecological development [8].

It is an effective solution to guide the main body of agricultural production and operation to fuel the conversion of agricultural waste mainly composed of straw. The development and utilization value of biomass energy has been proved in recent years [9]. For example, wood pellet fuels are of great value in the energy transition to replace fossil fuels [10]. The promotion of biomass clean heating in rural areas has a significant effect on emission reduction [11], which will help China achieve carbon neutrality goals and combat climate change [12].

What is the acceptance and willingness of consumers in the end product market to accept such products? It needs to be explored in depth. The consumers’ willingness is of great significance for motivating consumers to participate in the “Dual Carbon” strategy. Thus, it is necessary to understand it to realize low-carbon energy transition and to mitigate global climate change [13,14]. Research on rural residents’ willingness to consume (WTC) and willingness to spend (WTS) for BBF can provide insights for the formulation of biomass clean fuel promotion policies in rural areas [15]. WTC refers to whether rural residents are willing to consume BBF, as well as how much rural residents are willing to spend on BBF consumption. It can encourage the masses of rural residents to participate in the “Double Carbon” action, and lay a solid and reliable mass foundation for the realization of the “Double Carbon” goal.

The research on this aspect mainly focuses on the research on low-carbon consumption behavior. Low-carbon consumption behavior means that residents consciously implement a consumption behavior model of low energy consumption, low pollution, and low emissions in the process of daily consumption, including the whole process of purchasing, using management, and disposing of waste [16]. Residents’ low-carbon consumption behaviors include not only energy-saving behaviors such as water and electricity saving, but also consumption behaviors that help reduce carbon emissions, such as purchasing energy-saving products, participating in garbage recycling and sorting, and waste disposal [17]. The core of low-carbon consumption behavior is to reduce carbon emissions [18]. At present, the research on residents’ low-carbon consumption behavior mainly focuses on two topics: factors affecting residents’ low-carbon consumption behavior and policies to guide residents’ low-carbon behavior [16]. The former focuses on the deep psychological attribution and social environmental impact of low-carbon consumption behavior, and its mechanism of action on low-carbon consumption behavior [19]. The latter focuses on formulating low-carbon consumption intervention policies, as well as studying the public acceptance and effectiveness of these intervention policies [20]. At present, low-carbon consumption behaviors in China are more altruistic behaviors [21]. In the absence of personal incentives, low-carbon consumption behavior cannot be sustained for a long time. Therefore, the incentive of low-carbon consumption behavior of rural residents also needs to find an entry point.

The contribution of this research lies in the context of the “carbon neutrality” goal proposed by the Chinese government; while also facing serious air pollution problems, it integrates the influencing factors and intervention measures of rural residents’ low-carbon consumption behavior, and uses regression analysis to determine the behavior-driven mechanism of rural residents. To study the key factors of residents’ low-carbon consumption behavior, formulate long-term intervention measures for the government and enterprises, and provide reference and guidance for guiding residents’ daily low-carbon consumption behavior. In addition, the use of BBF will be an important complement to natural gas in the current European energy crisis caused by the wars in Russia and Ukraine. In the context of global warming, it is of great practical significance to let more consumers know about BBF with “carbon neutrality” properties. Research on “carbon neutrality” energy consumption driven by consumer behavior in different contexts has important reference value for promoting BBF in the current global energy crisis and environmental crisis.

Based on the analysis of the literature and the survey and interview data obtained in the survey, we propose the following hypothesis.

**Hypothesis** **1** **(H1).** *Individual characteristics of rural residents will affect their WTC and WTS for BBF to replace coal.*

**Hypothesis** **2** **(H2).** *Rural residents’ household characteristics will affect their WTC and WTS for BBF to replace coal.*

**Hypothesis** **3** **(H3).** *The relevant policy support cognition of the government has a significant positive impact on the WTC and WTS of rural residents.*

**Hypothesis** **4** **(H4).** *The more significant the cognitive characteristics of rural residents, the more positive the impact on their WTC and WTS.*

## 2. Materials and Methods

### 2.1. Survey Region

There are great differences in resource endowments in different regions of China. Compared with south China, the economic level of north China is relatively low, the proportion of the rural population is relatively high, and the improvement of rural living environment needs to be further strengthened. Shandong Province, located in the North China Plain, is a traditional agricultural province with the largest rural population and a high concentration of residential areas. In recent years, it has faced serious air pollution problems caused by coal burning [22]. It is suitable for promoting the use of replacing coal with BBF. In recent years, Longkou City, Laizhou City and Zhaoyuan City have focused on promoting such projects. As of November 2020, the per capita annual disposable income of rural residents in these three cities was 25,477 Chinese Yuan (CNY), 24,021 CNY and 24,591 CNY, respectively [23,24,25,26,27]. Through the research, it can provide reference for the promotion of BBF in other rural areas of Shandong Province and other provinces and cities in China. Therefore, this study selected these cities as the sampling areas.

### 2.2. Questionnaire and Variables

There are two dependent variables, WTC and WTS. In the actual investigation process, the first question is the WTC part. The question is designed as: “If the coal is banned to use in order to support the country’s carbon neutrality strategy and reduce air pollution, and it is recommended that you use BBF, would you be willing to consume it?” The answers are “1, yes; 0, no”. 

If the respondents answer yes, they enter the WTS session. In the WTS section, respondents will be directly asked: “With reference to the current annual coal consumption, under the guarantee of the current lifestyle, assuming no external subsidies, how much does the average person want to spend each year to buy BBF to replace coal?”. During the investigation, the investigators will make a special explanation: 2 tons of BBF can replace 1 ton of coal. Referring to the current average consumption cost of BBF in China’s rural areas, the average per capita consumption of biomass briquette is about 450 CNY/year (survey data, the average consumption of BBF per person in local rural areas is about 1 ton/year and the price is 450 CNY/ton). We also refer to the study of Liu and Sheng [28]. This question is also not closed-ended, and the answer is designed as: “A: 1–150 CNY, B: 151–300 CNY, C: 301–450 CNY, D: 451–600 CNY, E: 601–750 CNY, F: 751–900 CNY, G: the specific amount above 901 CNY”. The median of the questionnaire is 450 CNY, the lowest value is 1 CNY, and the highest value is 900 CNY. If it exceeds 900 CNY, directly fill in the buyer’s WTS amount. Finally, the interval value of the respondent’s WTS is obtained.

For the independent variables, this paper selects four dimensions. They are “rural residents’ individual characteristics”, “rural residents’ family characteristics” and “rural residents’ cognitive characteristics”. Much of the data came directly from respondents’ responses. According to the theory of peasant household behavior and the theory of planned behavior [29,30], the individual characteristics are selected as “gender”, “age”, “education level” and “income type”. The characteristics of family characteristics are selected as “the actual total population of the family”, “the family’s annual net income”, “the family’s planting area”, “the number of children in the family”, “the number of the elderly”, “whether there are village cadres in the family”, “the family’s annual coal consumption” and “energy preference”, a total of eight indicators. 

The characteristics of rural residents’ cognition were selected as “policy support cognition” [31], “health concept cognition” [32], “ecological environmental protection cognition” [33] and “resources sustainable use cognition” [34].

There are three questions in the rural residents’ cognitive characteristics. Rural residents’ environmental cognition is the basis of environmental governance. The behavior of rural residents is carried out under certain environmental cognition. The variables of rural residents’ cognitive characteristics reflect rural residents’ cognition and understanding of policy support cognition, health concept, ecological environment protection and resource regeneration. The questionnaire designed four questions to investigate: “If the government grants subsidies for the implementation of BBF to replace coal, are they willing to use them?”, “Do you think burning coal will affect the health of rural residents?”, “Do you think the use of BBF will effectively reduce carbon emissions and pollution to the atmospheric environment?” and “Do you think that BBF can fully reuse rural biomass waste and save energy?”. For any of the above questions, we record “disagree” as 1, “neutral” as 2 and “agree” as 3.

The definition and description of each variable are shown in Table 1.

### 2.3. Data Collection 

Before collecting data, we calculated the minimum sample size. According to the formula for calculating the sample size [35,36], we set the confidence interval to 90% and the degree of accuracy to 3%. The number of standard deviations that would include all possible values in the range is 4. By looking at the table, we observe t = 1.645 under the 90% confidence interval. The minimum sample size can be calculated as 128. This is basically consistent with the study of Adam et al. [37].

The data used in this study come from a field survey conducted in the Shandong Province of China from June to July 2022. This study used a combination of stratified sampling and random sampling for data collection. Respondents to this survey are permanent residents of the village and key members of their respective households. As a result, most of the respondents were able to make decisions about their family’s participation in BBF substitution projects.

The pre-survey will be conducted in Longkou City in June 2022. Based on the results of this pre-survey, the questionnaire was refined to ensure understandability and clarity. Thereafter, a revised questionnaire was applied and a formal survey was conducted in the survey area.

The survey for WTS uses the payment card method, which is the most basic guiding method of WTS. By directly providing a series of amounts for the respondents to choose, it can solve the questions that the respondents did not answer or protested, and improve the effective response rate of the survey. This study used a one-to-one survey method. In order to ensure that the interviewees can fully understand the replacing coal with BBF project, the researchers first spent 3–5 min to introduce the meaning and general content of the replacing coal with BBF to the respondents. It takes about 15 min to complete a questionnaire. The research team organized local rural residents to participate in this survey by contacting the local agricultural department.

Finally, we collected a total of 400 questionnaires, with 146, 124 and 130 from Longkou City, Laizhou City and Zhaoyuan City, respectively. Finally, after sorting and screening, a total of 365 valid questionnaires were obtained, with an effective rate of 91.25%.

### 2.4. Model

Respondents’ adoption of replacing coal with BBF is two independent decision-making processes: the first stage is the adoption decision stage, i.e., WTC; the second stage is the adoption degree, i.e., WTS. If respondents chose “no” to the second stage of behavior, the degree of adoption was unobservable. Therefore, there is a problem of self-selection bias in the respondents’ adoption of replacing coal with BBF, and the Heckman sample selection model needs to be used to deal with this problem [38]. Therefore, the following model needs to be constructed:

(1) $$Prob\left(\gamma =1\right)=\varphi \left(\beta_{0}+\sum_{i=1}^{n}\beta_{i}x_{i}\right)$$

The left side of the formula is the dependent variable, which represents the probability of an event occurring. In this article, it represents the probability that the respondents have the WTC (γ = 1, indicating that they have the WTC; γ = 0, indicating that they have no WTC). φ(·) on the right side of the formula is the cumulative normal distribution function, $\beta_{0}$ is a constant term, x_i_ is the n factors that affect the sample’s WTC and $\beta_{i}$ is the corresponding parameter to be estimated, reflecting the influence of explanatory variables on the WTC. After calculating the tendency of each sample’s WTC through the Probit model, this paper constructs a correction factor, which is as follows:

(2) $$\lambda =\varphi (\beta_{0}+\sum_{i=1}^{n}\beta_{i}x_{i})/[1-\varphi (\beta_{0}+\sum_{i=1}^{n}\beta_{i}x_{i})]$$

where λ is called the inverse Mills ratio or hazard ratio and ψ(·) and φ(·) are the density function and cumulative distribution function of the standard normal distribution, respectively.

In the second step, the inverse Mills ratio λ will be brought into the regression model as a control variable. The role of the inverse Mills ratio is to tell us whether the self-selectivity of WTC will have a significant impact on WTS. To examine the effect of WTC on WTS, we constructed a linear regression model with WTS as the dependent variable:

(3) $$y=\alpha_{0}+\alpha_{1}x_{1}+\sum_{i=2}^{n}(\alpha_{i}x_{i}+\omega \lambda +\varepsilon )$$

where $x_{1}$ is the core independent variable of WTC, $x_{i}$ is the control variable, λ is the inverse Mills ratio estimated according to formula 2, $\alpha_{0}$ is the regression constant term, $\alpha_{1}$, $\alpha_{i}$ and ω are the estimated parameters of the corresponding explanatory variables, $\alpha_{1}$ is the influence of WTC on WTS and ε is the error vector.

## 3. Results

### 3.1. Descriptive Analysis

The overall distribution of the samples is relatively uniform. Among the 365 questionnaires collected, 216 were male respondents, accounting for 59.2%, and 149 were female respondents, accounting for 40.8%. The gender ratio of the questionnaire was relatively balanced. In terms of age, 33 respondents were 20–30 years old, accounting for 9.04%; 58 respondents were 31–40 years old, accounting for 15.89%; 72 respondents were 41–50 years old, accounting for 19.73%; 132 people were aged 51–60, accounting for 36.16%; 63 people aged 61–70, accounting for 17.26%; 7 people were aged 71–80, accounting for 1.92%. Considering that the respondents of this questionnaire are rural residents, except for some younger villagers (20–31 years old) and older villagers (71–80 years old), the interviewed rural residents of other age groups are relatively evenly distributed, which indicates that the selected respondents are reasonable in terms of age distribution.

The economic and social development levels vary greatly between regions. Among the regions selected in this survey, wheat and corn are the main agricultural species, and the level of industrial development is moderate in China. As can be seen from Table 2, among the characteristics of the rural residents in this survey, 80% of the rural residents’ jobs are non-agricultural employment, and the average family planting area is 12.77 mu. It can be seen that the income from crop planting is not the main source of income for the rural residents in the surveyed areas, and the economic and social development levels vary greatly between regions.

Respondents’ WTC is relatively positive. When rural residents were asked “if the market provides them with high-quality BBF, would they be willing to use it instead of coal?”, 81.64% of the respondents would give a positive response. For rural residents who are willing to use it, 23% of the respondents are still worried about the price. This shows that if the cost is within the range that rural residents can afford, they will have a high enthusiasm for participating in the replacing coal with BBF project. For rural residents who are unwilling to participate in the project, 17.2% of the respondents said they did not want to participate in the project because they were accustomed to the current living energy structure, and 21.56% of the respondents said they were worried about the price and did not want to participate in the project. 

Most of the crop straw resources in the survey area are far from being fully exploited and utilized. As far as the current household energy usage is concerned, the average annual coal consumption of households in the survey area is 2.06 tons, and 71.56% of rural residents’ households consume no less than 2 tons of coal per year. Only 18.36% of households have an energy preference to use crop straw as fuel. 

### 3.2. Measurement Results 

We use Stata 16.0 software to calculate the sample, and use the Heckman sample selection model to calculate the quantitative analysis of WTC/WTS. The measurement results showed that the “Wald chi 2 = 774.40”, “*p* = 0.0000”, and the inverse Mills value was significant within the 90% confidence interval, indicating that the sample selection was biased. It is valid to choose the Heckman sample selection model. The measurement results are shown in Table 3.

According to Formula 3, after excluding the sample selection bias, the expected average WTS value of the interviewed rural residents in the survey area is 157.78 CNY/year. According to the family size, the annual WTS of each family is 913.55 CNY.

### 3.3. Results Analysis

It can be seen from Table 3 that the respondents’ “age”, “education level”, “work nature”, “annual household coal consumption”, “policy support cognition”, “health concept cognition”, “ecological environmental protection cognition” and “resources sustainable use cognition” have a significant impact on rural residents’ participation in the replacing coal with BBF project.

Among them, the “age” variable passed the significance test with 90% confidence level in both the WTC stage and WTS stage models, with coefficients of −0.05 and −0.76, respectively. This shows that the age of the respondents negatively affects rural residents’ WTC/WTS for replacing coal with BBF, that is, the younger the respondents, the more likely they are to participate in the project; additionally, the WTS is stronger. The hypothesis here is basically verified, that is, the younger the age, the higher the acceptance of biomass-to-coal and other clean heating policies, and the more positive the WTC and WTS.

The “educational level” variable did not pass the significance test at the WTC stage, but passed the significance test at the 99% confidence level at the WTS stage with a coefficient of 5.49. This indicates that respondents’ educational level, that is, years of education has no effect on their WTC, but has a significant positive effect on their WTS. In other words, other things being equal, the higher the education level of rural residents, the higher their WTC for using BBF. The possible reason is that rural residents with a higher education level have broader knowledge and vision, have a stronger understanding of clean heating policies such as replacing coal with biomass and are more WTS.

The “job category” variable passed the significance test with a 99% confidence level in the WTC stage with a coefficient of 3.37, but did not pass the WTS stage test. This indicates that the nature of the respondents’ work, i.e., non-farm employment, positively affects the respondents’ WTC, but has no significant effect on the respondents’ WTS. A possible reason is that rural residents with a high level of non-agricultural employment are less dependent on agricultural planting, more inclined to use BBF and their WTC is more active.

The variable “annual coal burning” did not pass the significance test in the WTC stage, but passed the significance test at the 95% confidence level in the WTS stage, with a coefficient of 16.01. This indicates that the annual coal consumption of the respondents’ households has little effect on their WTC, but will positively affect the respondents’ WTS. The possible reason is that the more coal burning households annually indicates that they have a greater demand for fuel, and the expenditure on coal purchases is relatively high. Therefore, they will pay more attention to the cost-effectiveness of fuel products when purchasing, and are more positive about the WTS of BBF.

The “policy support cognition” variable passed the significance test with 95% and 99% confidence levels in the WTC stage and WTS stage models, respectively, with coefficients of 1.74 and 30.28, respectively. This indicates that government support has a significant positive impact on respondents’ WTC and WTS. Policy support cognition, that is, the government grants financial subsidies to rural residents who use BBF to reduce rural residents’ participation cost. According to the survey, if the government provides subsidies, rural residents will tend to accept the project of replacing coal with BBF for their own economic interests. If the government does not provide subsidies, even if the cost of using BBF is slightly lower than that of traditional energy sources such as coal, rural residents may not choose BBF. The possible reason is that when rural residents use BBF, they also need to consider the price and performance of heating facilities, such as supporting stoves, and the expenditure in this regard is also a large expense for rural residents. Therefore, the financial support of the policy will play a positive guiding and stimulating role in the development, utilization and promotion of BBF.

The “health concept cognition” and “resources sustainable use cognition” in the rural residents’ cognitive characteristic variables passed the significance test at the 90% and 95% levels, respectively, in the WTC stage. Both had a significant positive impact on rural residents’ WTC. This indicates that the higher the cognition of rural residents’ health concept and the stronger understanding of resource regeneration, the more positive their WTC/WTS. The possible reason is that, on the one hand, with the improvement of living standards, rural residents pay more attention to their own health status and the requirements for the living environment. When rural residents perceive changes in their living environment and threaten their health and daily life, they will actively participate in the use of biomass to clean heating, and their WTS will also increase. The variable of “ecological environmental protection cognition” did not pass the significance test of the WTC stage, but passed the test of the WTS stage with a 99% confidence level, with a coefficient of 72.64. The reason may be that rural residents do not have enough understanding of the role and status of ecological civilization construction, and they do not have a deep understanding of the relationship between biomass energy utilization projects and ecological environmental protection. This also shows that strengthening the publicity of clean energy and enhancing rural residents’ cognition of ecological and environmental protection will be an important part of accelerating the replacement of coal with BBF.

## 4. Discussion

Based on the research, it is indeed necessary to take some measures to increase rural residents’ WTC in replacing coal with BBF. First, according to the actual situation in various places, it is recommended to increase the financial investment at the government level, set up special funds for projects and strengthen financial support services. According to the results, the average maximum purchase intention of rural residents in the survey area is 157.78 CNY/year. However, in the current survey area, there is a large gap between the WTS of rural residents and the actual cost of BBF. In order to improve the sustainability of the project, financial support mechanisms such as demand response-based subsidies need to be established. Demand response is considered a potentially cost-effective means [39]. Therefore, due to the regional differences between different regions, it is also suggested that the government should give full play to the regional advantages and formulate appropriate rural biomass energy clean heating subsidy policies according to local conditions.

Giving full play to the market-oriented long-term mechanism and rationally calculating input and output will effectively stimulate rural residents’ WTC. According to the survey, although only 18.36% of the rural residents in the study area are using crop straw fuel, nearly 81.64% of the respondents expressed their WTC in the replacing coal with BBF project. This shows that it is expected that 63.28% of rural residents in the survey area will consume BBF in the future. This also means that in order to meet the needs of these rural residents, combined with the WTS survey data, the government still needs to invest huge amounts of project subsidies. This clearly reflects the enormous pressure on government finances and a severe shortage of funds. It can be seen that if only relying on the investment participation of the government and rural residents, the process of improving the rural ecological environment, especially the straw briquetting project to replace coal, will be relatively slow. However, in view of the huge ecological and economic benefits of the project, it is necessary to comprehensively consider the production cost, the market price and the WTS price of rural residents [40]. To this end, it is recommended to use statistical analysis methods to study and propose an effective way of rural environmental governance that combines the government and the market. This suggestion is also consistent with the suggestion made by Teng et al. [41]. Therefore, it is recommended to use various funding sources, encourage the active participation of social organizations such as the government, enterprises and rural areas, give full play to the long-term market-oriented mechanism, and jointly establish a commodity market system with balanced supply and demand and a project development mechanism with a balance of profit and loss, so as to ensure the project’s sustainable development.

We also need to pay attention to some negative factors that restrict the development of the project. The survey data showed that the age of rural residents had a great negative impact on their WTC/WTS. It is estimated that in 2057, China’s population over the age of 65 will reach a peak of 425 million, accounting for 32.9–37.6% of the total population [42]. Massive and rapid population ageing will present economic, social and governance challenges [43]. Especially in rural areas, with the massive migration of young and young laborers, the proportion of the elderly population has increased significantly, which will lead to an increasingly serious problem of lack of talents for rural revitalization [44]. The influence of the aging of the rural population on the carbon emissions of rural households will also expand significantly, which is basically consistent with the conclusion of Fan et al. [45]. Therefore, scientifically guiding and motivating the elderly in rural areas to actively participate in carbon emission reduction and air pollution control is an issue worthy of attention in the development process of China’s development of the “double carbon” goal.

Furthermore, it is interesting to find in our study that eco-environmental cognition has a positive effect on rural residents’ WTS, but not significantly on their WTC. The possible reason is that the rural areas are limited by factors such as the level of scientific and technological education, which makes rural residents less accepting of ecological and environmental protection. However, with the improvement of living standards, rural residents pay more attention to their own health and their living environment requirements will gradually increase. According to the suggestions put forward by Yao and Fang, embed big data technology into rural ecological governance to further improve rural residents’ ecological and environmental protection cognition, so as to realize the modernization of rural ecological governance in the era of big data [46]. It is necessary to go deep into the rural grassroots, establish the concept of ecological development ideologically, benefit the people from the dividends of ecological development and make green and low-carbon development a social consensus [47].

The behavior-driven mechanism of rural residents’ participation in “carbon neutrality” is the endogenous driving force for rural energy improvement to achieve the goal of “carbon neutrality”. As consumers, rural residents are the main body in the industrial chain that converts biomass resources into clean energy [48]. The construction of a rural clean energy system must be closely integrated with addressing the needs of rural residents. China’s rural areas have been lifted out of poverty as a whole, but there is a large gap in energy development between urban and rural areas [49], and energy is relatively poor, especially in rural clean energy development is seriously lagging behind [50]. Therefore, it is necessary to fully study the behavior-driven mechanism of rural residents’ participation in “carbon neutrality”, so that rural residents can “afford, use well, and use for a long time” energy, in order to realize the sustainable development of rural resources and energy coupling.

The above research conclusions have important policy implications. With the background that the current rural ecological environment governance is still dominated by government regulations, it is necessary to gradually strengthen the implementation of rural biomass coal substitution. It is necessary to adjust the leading direction and intensity of the policy flexibly and effectively in light of the actual situation. First of all, government subsidies for biomass energy clean heating projects should be increased to reduce the participation cost of rural residents. Financial support services can be strengthened through the use of financial, taxation and other means to set up special funds for projects. Secondly, market entities should be encouraged and guided to innovate in the direction of low-carbon development, expand the pillar industries of the bio-economy and increase the market supply of biomass fuel products to meet consumer demand. Thirdly, it can be promoted by the connection between high-quality ecological education resources and rural areas through digital technology and other means. In the national education system, emphasis can be placed on ecological civilization education that includes comprehensive utilization of biomass energy and green and low-carbon development. We should increase the publicity of the importance of low-carbon energy transition in rural areas, improve rural residents’ perception of global climate change and then increase rural residents’ participation in replacing coal with biomass projects. Finally, we should organize and guide the elderly in rural areas to participate in green and low-carbon activities. Adopting these recommendations may help incentivize rural residents to participate in carbon-neutral actions, accelerate the rural energy transition process and further contribute to the realization of the “double carbon” goal.

## 5. Conclusions

This paper takes as a starting point the data of 365 rural household questionnaires in three county-level cities in Shandong Province, taking the replacing coal with BBF project as an example, using the Heckman sample selection model to estimate the rural residents’ WTC and WTS, and identified potential factors influencing rural residents’ decision making. The research results show that 81.64% of the respondents are willing to consume the BBF, but there is still a big gap between the average WTS and the actual cost. This study also explored the relationship between the factors affecting rural residents’ WTC and WTS for BBF, such as rural residents’ individual characteristics, family characteristics, and rural residents’ cognitive characteristics. Finally, we propose policy recommendations for behavior-driven mechanisms of consumer participation in the “carbon neutrality” strategy.

However, this study has certain limitations. First, the data analyzed in this study were collected from only three counties and cities in Shandong Province, so the results of this study may not be applicable to the development of all regions. Therefore, if the scope of the investigation is expanded, and relevant research is carried out according to the actual development situation according to local conditions, the obtained research results may enrich the results of this research field. Secondly, the convenience of transportation in rural areas, the construction of biomass fuel supporting facilities and the maturity of the biomass energy market may also be important factors that affect rural residents’ participation in decision making. Therefore, these factors will be further developed in follow-up research.

## Figures and Tables

**Table 1:** The variables’ definition and description.

Variable		Code	Description	Options
Dependent Variable		WTC	Would you like to consume the BBF to replace coal?	1 = yes, 0 = no
		WTS	How much are you willing to spend on BBF to replace coal? Based on the amount spent per person per year.	A: 1–150; B: 151–300; C: 301–450; D: 451–600; E: 601–750; F: 751–900; G: the specific amount above 901
Independent Variable	Individual characteristics	Gender	Respondent’s gender	1 = male, 0 = female
		Age	Respondent’s age	Number
		Education	Actual years of education	Number
		Job category	Whether it is non-agricultural employment	1 = yes, 0 = no
	Family characteristics	Population	Actual household size	Number
		Annual income	Average annual household income	Number
		Planting area	The actual planting area of the family	Number
		Child	Number of children under 10	Number
		Elder	Number of seniors over 65 years old	Number
		Village cadre	Are there any village officials at home?	1 = yes, 0 = no
		Annual coal consumption	Average annual coal consumption by households	Number
		Energy preference	Does the household use straw-based fuel?	1 = yes, 0 = no
	Rural residents’ cognitive characteristics	Policy support cognition	If the government grants subsidies for the implementation of BBF to replace coal, are they willing to use them?	1 = yes, 0 = no
		Health concept cognition	Do you think burning coal will affect the health of villagers?	Disagree = 1, Neutral = 2, Agree = 3
		Ecological environmental cognition	Do you think the use of BBF will effectively reduce carbon emissions and pollution to the rural atmospheric environment?	Disagree = 1, Neutral = 2, Agree = 3
		Resources sustainable use cognition	Do you think that BBF can make full use of rural straw and other wastes and save energy?	Disagree = 1, Neutral = 2, Agree = 3

**Table 2:** Descriptive statistics for variables.

Category	Code	Value Setting	Mean Value	Std. Dev.
Dependent Variable	WTC	Yes = 1, No = 0	0.82	0.39
	WTS	Unit: CNY	163.60	688.71
Individual characteristics	Gender	Male = 1, Female = 0	0.59	0.49
	Age	Amount	50.00	12.01
	Educational level	Amount	7.21	2.99
	Job category	Yes = 1, No = 0	0.80	0.40
Family characteristics	Population	Amount	5.79	2.16
	Annual income	Amount	32,312	24,110
	Planting area	Unit: Mu	12.77	40.95
	Child	Amount	1.22	0.41
	Elder	Amount	0.72	0.78
	Village cadre	Yes = 1, No = 0	0.19	0.40
	Annual coal consumption	Unit: t	2.06	0.83
	Energy preference	Yes = 1, No = 0	0.18	0.39
Rural residents’ cognitive characteristics	Policy support cognition	Yes = 1, No = 0	0.51	0.50
	Health concept cognition	Score	1.85	0.85
	Ecological environmental cognition	Score	1.58	0.78
	Resources sustainable use cognition	Score	1.79	0.79

**Table 3:** Measurement results.

Category	Variable	Stage 1 (WTC)		Stage 2 (WTS)	
		Coef.	St.Err.	Coef.	St.Err.
Individual characteristics	Gender	0.08	0.46	−3.02	8.28
	Age	−0.05 *	0.03	−0.76 *	0.43
	Educational level	0.05	0.10	5.49 ***	1.59
	Job category	3.37 ***	0.64	22.57	22.45
Family characteristics	Population	0.04	0.10	1.29	1.91
	Annual income	−0.00	0.00	0.00	0.00
	Planting area	0.02	0.05	0.04	0.11
	Child	−0.91	0.72	−6.43	14.43
	Elder	−0.44	0.29	−5.08	5.38
	Village cadre	0.85	0.80	−4.73	14.40
	Annual coal consumption	−0.11	0.31	16.01 **	6.47
	Energy preference	−0.33	0.46	−12.92	11.64
Rural residents’ cognitive characteristics	Policy support cognition	1.74 **	0.76	30.28 ***	9.43
	Health concept cognition	0.90 *	0.47	39.49 ***	6.52
	Ecological environmental cognition	0.27	0.75	72.64 ***	6.27
	Resources sustainable use cognition	1.02 **	0.40	27.86 ***	6.20
	_cons	−0.59	2.14	−142.07 ***	47.63

Note: *, ** and *** mean significance at the statistical level of 10%, 5% and 1%, respectively.

## Data Availability

The data presented in this study are available on request from the corresponding author.
